# The Putative GATA Transcription Factor *Sb*GATA22 as a Novel Regulator of Dhurrin Biosynthesis

**DOI:** 10.3390/life14040470

**Published:** 2024-04-03

**Authors:** Viviana C. Rosati, Alicia A. Quinn, Roslyn M. Gleadow, Cecilia K. Blomstedt

**Affiliations:** 1 School of Biological Sciences, Monash University, Wellington Road, Clayton, VIC 3800, Australia; viviana.rosati@tozerseeds.com (V.C.R.); alicia.quinn1@monash.edu (A.A.Q.); ros.gleadow@monash.edu (R.M.G.); 2 Queensland Alliance for Agriculture & Food Innovation, The University of Queensland, St Lucia, QLD 4072, Australia

**Keywords:** cyanogenesis, cyanogenic glucosides, GATA transcription factor, regulation, specialized metabolites

## Abstract

Cyanogenic glucosides are specialized metabolites produced by over 3000 species of higher plants from more than 130 families. The deployment of cyanogenic glucosides is influenced by biotic and abiotic factors in addition to being developmentally regulated, consistent with their roles in plant defense and stress mitigation. Despite their ubiquity, very little is known regarding the molecular mechanisms that regulate their biosynthesis. The biosynthetic pathway of dhurrin, the cyanogenic glucoside found in the important cereal crop sorghum (*Sorghum bicolor* (L.) Moench), was described over 20 years ago, and yet no direct regulator of the biosynthetic genes has been identified. To isolate regulatory proteins that bind to the promoter region of the key dhurrin biosynthetic gene of sorghum, *SbCYP79A1*, yeast one-hybrid screens were performed. A bait fragment containing 1204 base pairs of the *SbCYP79A1* 5′ regulatory region was cloned upstream of a reporter gene and introduced into *Saccharomyces cerevisiae*. Subsequently, the yeast was transformed with library cDNA representing RNA from two different sorghum developmental stages. From these screens, we identified *Sb*GATA22, an LLM domain B-GATA transcription factor that binds to the putative GATA transcription factor binding motifs in the *SbCYP79A1* promoter region. Transient assays in *Nicotiana benthamiana* show that *Sb*GATA22 localizes to the nucleus. The expression of *SbGATA22*, in comparison with *SbCYP79A1* expression and dhurrin concentration, was analyzed over 14 days of sorghum development and in response to nitrogen application, as these conditions are known to affect dhurrin levels. Collectively, these findings suggest that *Sb*GATA22 may act as a negative regulator of *SbCYP79A1* expression and provide a preliminary insight into the molecular regulation of dhurrin biosynthesis in sorghum.

## 1. Introduction

Cyanogenic glucosides (CNglcs) are plant specialized metabolites. Derived from amino acids, they enable a cyanide group to be stably stored as a non-toxic compound via the glycosylation of a cyanohydrin. Over 3000 species of higher plants produce CNglcs, including important crop species such as barley, sorghum, clover, cassava, apples, and almonds, with their biosynthesis tightly linked to ontogeny, tissue type, and environmental factors, including light, water, and nitrogen availability [1,2]. Over 60 CNglcs have been identified, and although all can be broken down to release hydrogen cyanide gas (HCN), the exact compound produced by each species differs due to the different amino acids and sugars used in their biosynthesis [1]. CNglcs were first characterized for their role in herbivore defense, as the released HCN binds to cytochrome c oxidase in the mitochondria, inhibiting cellular respiration [3,4]. More recently, alternative turnover pathways of CNglcs that release ammonia and carbon dioxide for plant growth, rather than HCN for defense, have also been identified [5,6,7], opening up the possibility of a role for CNglcs in primary (in addition to secondary) metabolism.

The concentration of CNglcs found in plant tissues varies between species, as does the site of synthesis [1]. CNglc concentrations are highly dynamic, generally decreasing as the plant matures, although the opposite pattern has been observed in some species [1,8]. Biotic and abiotic factors, such as herbivory [9], pathogen attack [10], light [8], drought [11], and nitrogen application [12], can also induce or repress CNglc biosynthesis in cyanogenic plants.

Cyanogenic glucosides can be problematic when cyanogenic plants are used for human and animal consumption. In sorghum, forage that is high in CNglcs can cause chronic or acute cyanide poisoning in ruminants, such as sheep and cattle [13]. The plasticity of CNglcs complicates predicting forage toxicity, particularly during periods of drought [11,14]. While removing the capacity of these crops to produce CNglcs appears to be a solution, their putative roles in nitrogen remobilization and stress tolerance may result in reduced growth rates and yields in acyanogenic lines [6,7,12,15,16,17].

The first CNglc biosynthetic pathway to be elucidated was the synthesis of dhurrin in sorghum (*Sorghum bicolor* (L.) Moench). Dhurrin is found in all vegetative tissues of sorghum but not in the mature grain [18]. Dhurrin concentrations are highest when plants are young, peaking between three to four days post-germination before decreasing in all tissues [19,20]. The dhurrin biosynthetic pathway begins with an L-tyrosine precursor and involves three enzymes coded for by three structural genes: two cytochromes P450, *SbCYP79A1* and *SbCYP71E1*, and the glycosyltransferase *SbUGT85B1* [21,22,23]. CYP79s are signature enzymes of these pathways, with *SbCYP79A1* being the rate-limiting enzyme of dhurrin biosynthesis in sorghum [20]. In sorghum, the three dhurrin biosynthetic genes are clustered on chromosome one [1,24].

Currently, little is known about the molecular regulation of dhurrin biosynthesis, although genome-wide demethylation has been found to affect *SbCYP79A1* expression in sorghum [25]. The majority of sorghum studies have focused on dhurrin regulation at the physiological level or analyzed gene expression without identifying the drivers of this expression [18,26,27,28]. The manipulation of dhurrin production has involved knocking out genes of the biosynthetic pathway rather than altering regulation [29,30]. Investigations into the regulation of CNglc production in almonds have identified a bHLH transcription factor that regulates the transcription of two cytochrome P450 genes, *PdCYP79D16* and *PdCYP71AN24,* involved in the biosynthesis of amygdalin [31]. In *Lotus japonicus*, methyl jasmonate has been shown to induce the expression of genes involved in the synthesis of the CNglc lotaustralin, and a bHLH transcription factor has been identified that controls this response [32]. While dhurrin is also believed to be regulated at the transcriptional level in sorghum [18,20] and methyl jasmonate has been shown to induce *SbCYP79A1* expression and increase dhurrin concentration in leaves [28], to date, no direct regulator of the dhurrin biosynthetic genes has been reported.

In this study, we used yeast one-hybrid screens to identify candidate regulators of the key dhurrin biosynthetic gene *SbCYP79A1*. Yeast one-hybrid (Y1H) screening is a valuable technique used to identify physical interactions in vivo between a DNA sequence of interest (known as the “bait” sequence) and a protein (the “prey”) [33]. The bait sequence is placed upstream of a reporter gene, and protein-coding sequences from a cDNA library are fused to a yeast GAL4 transcriptional activation domain. Positive interactions between the bait and prey are detected by reporter gene expression in the yeast *Saccharomyces cerevisiae*. This enables the identification of novel DNA–protein interactions and provides an advantage over methods such as ChIP-Seq, as it does not require the prior identification of DNA-binding proteins. To the best of our knowledge, this is the first time this strategy has been applied to a gene involved in sorghum CNglc biosynthesis.

As cis-regulatory elements that may be important in *SbCYP79A1* regulation are largely unknown, a native promoter fragment of 1204 bp was used as bait rather than an artificial run of repeated binding motifs. Where there is little or no information on possible transcription factors, the use of a complex, longer-length promoter sequence of the gene of interest has advantages over short (typically < 30 bp) repeat sequences of known transcription factor binding sites, as it allows for a broader identification of possible regulatory proteins [34]. Specific gene promoters up to 2 kb in length have been used as bait sequences in large-scale Y1H screens intended for regulatory network analyses [35,36]. cDNA libraries from two early developmental stages were constructed, as the synthesis of dhurrin is at its maximum during early seedling growth [8,19]. The first screen used seeds that had been imbibing for 24 and 48 h, corresponding to the developmental stage prior to maximum dhurrin concentration. The second screen used coleoptile tissue from seedlings three days post-germination, corresponding to when dhurrin concentration is at a maximum. From these screens, positive colonies were selected, and their inserts were characterized, resulting in the identification of a putative GATA transcription factor, *Sb*GATA22. The interaction of *Sb*GATA22 with putative GATA-binding motifs in the *SbCYP79A1* promoter region is reported. The transcript abundance of *SbGATA22* during sorghum development and in response to nitrogen application is also detailed. As nitrogen application is known to increase the expression level of dhurrin biosynthesis genes in mature plants [20], we tested whether *Sb*GATA22 may also be involved in controlling dhurrin biosynthesis in response to nitrogen application.

This study provides a preliminary insight into the transcriptional control of dhurrin biosynthesis in sorghum. Uncovering the molecular networks regulating CNglc production may enable crop toxicity predictions that are more accurate than the current methodologies permit and may also allow for CNglc production to be manipulated in ways that do not compromise yield or stress tolerance.

## 2. Materials and Methods

### 2.1. Yeast One-Hybrid Screen: Construction of the CYP79A1 Promoter–AbAi Reporter (Bait) Plasmids

A native *SbCYP79A1* (*Sb01g001200*) promoter region (1204 bp upstream of the transcription start site) (Supplementary Figure S1) was synthesized by GenScript (Piscataway, NJ, USA) and cloned into pUC57. The native promoter:pUC57 vector was digested with KpnI and SalI, and the resulting promoter insert sequence was ligated into the vector pAbAi using a 1:1 vector:insert ratio. This produced the vector pAbAi-Native. The construct was electroporated into DH5α *E. coli* cells and selected by ampicillin resistance (50 μg mL^−1^). The construct was purified from *E. coli* using the *Accuprep* Plasmid Mini Extraction Kit (Bioneer Corporation, Daejeon, Republic of Korea) and sequenced to ensure no mutations were present. The vector was linearized with BstBI, and 1 μg was transformed into *S. cerevisiae* Y1H Gold (Takara Bio Inc., Shiga, Japan) using the small-scale yeast transformation method as described in the Matchmaker Yeast One-Hybrid Library Screening System manual (Takara Bio Inc., Shiga, Japan). Cells were spread onto synthetic dropout media lacking uracil (SD/-Ura) and allowed to grow for three days at 30 °C. Colonies were analyzed by colony PCR using the Matchmaker Insert Check PCR Mix 1 (Takara Bio Inc., Shiga, Japan) to identify clones with correctly integrated vectors.

To test the bait strain for autoactivation of the *AUR1-C* reporter gene, yeast transformed with the pAbAi-Native vector was spread onto SD/-Ura media containing the antibiotic Aureobasidin A (AbA) between 100 and 200 ng mL^−1^. Plates were incubated for five days at 30 °C to determine the concentration of AbA required to inhibit autoactivation of the AbA reporter gene by endogenous yeast transcription factors.

### 2.2. Plant Material

Two cDNA libraries were constructed and screened using plant tissue from different developmental stages:

Library 1 (Imbibed seed): Seeds of *Sorghum bicolor* (L.) Moench line BTx623 were sterilized and germinated in Petri dishes containing filter paper wetted with sterile MilliQ H_2_O. Petri dishes were placed in the dark at 28 °C and imbibed for either 24 or 48 h, at which point, the seeds with emerging radicles (24 h) or radicles and emerging coleoptiles (48 h) were snap-frozen in liquid nitrogen and stored at −80 °C.

Library 2 (Coleoptiles): Seeds underwent the same process as above but were germinated for three days, at which point, the tips of the coleoptiles (1 cm in length) were cut off; the coleoptile tips were pooled, snap-frozen in liquid nitrogen, and stored at −80 °C. The remainder of the coleoptiles, seeds, and radicles were discarded.

### 2.3. RNA Extraction and cDNA Library Construction

Seeds that had imbibed for 24 h and 48 h were pooled, and 500 mg of the tissue was ground to a fine powder using a mortar and pestle chilled on dry ice. The same procedure was undertaken for the coleoptile tissue. Total RNA from both samples of ground tissue was extracted using a LiCl/phenol extraction method [37]. RNA integrity was analyzed using a 2100 Bioanalyzer (Agilent Technologies, Santa Clara, CA, USA), and samples with an RNA integrity number (RIN) greater than 9 were used for mRNA extraction. mRNA was extracted using the Dynabeads mRNA Purification Kit (Invitrogen, Waltham, MA, USA) and then treated with RQ1 RNase-free DNase (Promega, Madison, WI, USA) for 30 min at 37 °C. mRNA was extracted from the DNase mix with 1:1 phenol:chloroform; precipitated from the aqueous phase with a 1/10 volume of 3M sodium acetate, pH 5.2, and 2.5 volumes of ethanol; and re-suspended in 5 μL of sterile MilliQ H_2_O.

### 2.4. cDNA Synthesis for Library Construction

First-strand SMART cDNA, with flanking end sequences homologous to the vector pGADT7-Rec, was synthesized from 1 μg of mRNA extracted from imbibed seed or coleoptiles using the Matchmaker Yeast One-Hybrid Library Screening System (Takara Bio Inc., Shiga, Japan). Double-stranded cDNA was generated and amplified by long-distance PCR using the Advantage 2 PCR Kit (Takara Bio Inc., Shiga, Japan) with an oligo-dT primer. The concentration and size of the resultant double-stranded cDNA were analyzed on a 1.2% agarose/EtBr gel alongside a 1 kb ladder. Samples were purified using CHROMA SPIN +TE-400 Columns (Takara Bio Inc., Shiga, Japan), precipitated via ethanol precipitation, and re-suspended in 20 μL sterile MilliQ H_2_O.

### 2.5. cDNA Library Screening

The yeast one-hybrid cDNA library construction and screening were performed using the Matchmaker Yeast One-Hybrid Library Screening System (Takara Bio Inc., Shiga, Japan). The Y1H pAbAi-Native bait strain was co-transformed with cDNA and pGADT7-Rec that had been linearized with SmaI (Takara Bio Inc., Shiga, Japan). This allowed for the cDNA inserts and vector to recombine in the yeast cells, placing the cDNA “prey” inserts downstream of a GAL4 activation domain. Co-transformation was undertaken using the large-scale yeast transformation method, as described in the Matchmaker Yeast One-Hybrid Library Screening System manual. To calculate the number of clones screened, 100 μL of the transformation reaction was diluted to 1/10, 1/100, 1/1000, and 1/10,000 and spread on synthetic dropout media lacking leucine (SD/-Leu). The remainder of the transformation reaction was spread onto SD/-Leu/AbA^100^ media and incubated at 30 °C for 5 days. After 5 days, the number of clones screened was calculated as

$$clones\;screened=\left(\frac{cfu}{ml}on\frac{SD}{-Leu}\right)\times \left(dilution\;factor\right)\times (resuspension\;volume\;\left(15mls\right))$$

Colonies on the SD/-Leu/AbA^100^ plates greater than 2 mm in diameter were re-streaked onto fresh media and grown at 30 °C for three days. Healthy colonies were analyzed by yeast colony PCR using the Matchmaker Insert Check PCR Mix 2 (Takara Bio Inc., Shiga, Japan). Products were purified using the *Accuprep* PCR/Gel Purification Kit (Bioneer, Corporation, Daejeon, Republic of Korea) and sequenced with a T7 primer. Vectors containing inserts of interest were rescued and transformed into DH5α *E. coli* via electroporation and selected by ampicillin (50 μg mL^−1^). The construct was purified from *E. coli* using the *Accuprep* Plasmid Mini Extraction Kit (Bioneer, Corporation, Daejeon, Republic of Korea) and re-sequenced to confirm the cDNA insert sequence.

Following the identification of *Sb*GATA22 in Screens 1 and 2, confirmation of its binding to the putative GATA transcription factor binding motifs in the *SbCYP79A1* promoter region was undertaken. A mutant *SbCYP79A1* (*Sb01g001200*) promoter region with all putative GATA transcription factor binding sites mutated from GAT into GTA (Supplementary Figure S1) was synthesized by GenScript (Piscataway, NJ, USA) and cloned into pUC57. The pAbAi-Mutant constructs were transformed into *S. cerevisiae* Y1H Gold (Takara Bio Inc., Shiga, Japan) and tested for autoactivation of the bait sequence as described above for the pAbAi-native construct. Y1H pAbAi-Mutant strains were then transformed with 1μg of pGADT7-Rec vector that contained the full-length *Sb*GATA22 insert from Screen 1 (Supplementary Figure S2) and selected on SD/-Leu media using the small-scale yeast transformation method. Colonies were analyzed by yeast colony PCR using the Matchmaker Insert Check PCR Mix 2 (Takara Bio Inc., Shiga, Japan) to ensure the correct vector was present. The Y1H *Sb*GATA22-Mutant strain was spread onto SD/-Leu/AbA^100^ media, and growth was compared with that of the strain harboring the native promoter sequence (*Sb*GATA22-Native) after three days at 30 °C.

### 2.6. Expression of SbGATA22 during Development

Expression of *SbGATA22* was analyzed over 14 consecutive days of sorghum development. *Sorghum bicolor* (L.) Moench line BTx623 was grown in the greenhouse complex at Monash University, Clayton, Vic., Australia, in August 2018. The greenhouse received natural light, with temperatures ranging from 18 °C to 28 °C, with an average of 26 °C/20 °C day/night. Seeds were germinated and grown in punnet trays on Debco seed-raising substrate (Debco, Bella Vista, NSW, Australia) and perlite (2:1 *v*/*v*). Plants were watered to saturation every second day to ensure water was never limited. Harvesting occurred daily from 1 day post-germination (dpg) to 14 dpg and commenced at 11 am each day to minimize possible circadian differences. Tissues from each plant were separated into leaf, stem, and root tissue, except for the first four days, where shoot tissues (the coleoptile on days 1–2; the leaf and stem on days 3–4) were harvested together due to the small size of the plants. For HCNp analysis, approximately 20 mg was taken from the coleoptile tip (days 1–2), the first fully unfurled leaf (days 3–14), the base of the stem (days 3–14), and the root tips (days 1–14) for 10 replicates. The remainder of the tissue was snap-frozen in liquid N for six of the ten replicates and stored at −80 °C for qPCR analysis.

### 2.7. Gene Expression in Response to Nitrogen Application

Plants were grown as detailed above in individual pots 20 cm in diameter. At 5 weeks post-germination, a baseline harvest was undertaken (Time 0). Following the Time 0 harvest, plants were watered daily with 400 mL of water (control) or 25 mM of KNO_3_ (treatment). Further harvests were completed after two days (Time 1) and five days (Time 2) of KNO_3_ application. At each time-point, tissue was harvested from 10 plants for HCNp analysis by taking three leaf discs (4 mm in diameter) from the youngest fully unfurled leaf and approximately 20 mg of tissue from the base of the stem and the root tips. For gene expression analysis, 200 mg of the same tissues from three plants was collected and snap-frozen in liquid N and then stored at −80 °C.

### 2.8. HCNp Analysis

Hydrogen cyanide potential (HCNp) was determined using approximately 20 mg of fresh leaf, stem, or root tissue, following Gleadow et al. [38]. HCNp is the total amount of HCN produced by the hydrolysis of the entire contents of endogenous cyanogenic glucosides (CNglcs), as achieved by adding exogenous β-glucosidase (β-D-Glucoside glucohydrolase, G4511, Sigma-Aldrich Burlington, MA, USA). The tissue sample, along with a degradative enzyme, was sealed in a glass vial and subjected to a freeze–thaw cycle to disrupt the tissue membranes. The HCN produced was captured as NaCN in a 1M NaOH solution within the vial and measured using a colorimetric assay [38]. This assay is used as a proxy for dhurrin, such that each mg of HCN is equivalent to 11.5 mg of dhurrin in the plant tissue. Comparison of this method with the direct measurement of dhurrin using LCMS has shown the results to be highly concordant [14]. Following the assay, all tissue was dried and accurately weighed to calculate mg HCN g^−1^ dry mass.

### 2.9. Quantitative PCR (qPCR)

RNA was extracted from the frozen leaf, stem, and root tissues using a Sigma Spectrum Plant Total RNA kit (Sigma-Aldrich, Burlington, MA, USA). On-column DNase digestion was carried out using RQ1 RNase-Free DNase (Promega, Madison, WI, USA) for all samples. RNA concentration was determined using a NanoDrop spectrophotometer (ND-1000, Thermo Fisher Scientific, Waltham, MA, USA), and the quality was checked by running the RNA on an 8% formaldehyde gel. cDNA was synthesized from the total RNA using a SuperScript III First-Strand Synthesis System for RT-PCR (Invitrogen, Waltham, MA, USA) and an oligo-dT primer. Transcript levels of *SbCYP79A1* (*Sb01g001200*) and *SbGATA22* (*Sb10g022580*), normalized to *Ubiquitin* (*Sb01g030340*) [14], were determined by quantitative PCR (qPCR) for each tissue (leaf, stem, and root). Relative expression levels of *nitrate reductase* (*Sb07g022750*) were also measured for the nitrogen experiment. Previous research normalizing gene expression to two reference genes [25] has shown no significant differences when using both *Ubiquitin* and *Actin* (*Sb01g010030*) in conjunction or when using only *Ubiquitin* [12,28]. An EpMotion 5075 Robot (Eppendorf, Hamburg, Germany) was used to set up the 384 well plates, and qPCR reactions were performed using a Light Cycler 480II (Roche, Basel, Switzerland) with cycling parameters as follows: initial denaturation: 10 min, 95 °C; denaturation: 15 s, 95 °C; annealing: 15 s, 66/63 °C; extension: 15 s, 72 °C (acquired at end of extension), for 40 cycles. Melt profile analysis was undertaken from 60 to 99 °C rising by 1 °C and waiting for 5s at each step. SensiMix SYBR Green No ROX kit (Bioline, Meridian Bioscience, Cincinnati, OH, USA) was used for the qPCR, as per the manufacturer’s instructions, with forward and reverse primers specific to a region in the 3′ end of each gene sequence used for amplification (Supplementary Table S1). Each biological replicate (*n* = 6 for developmental stages; *n* = 3 for nitrogen experiment) was run in triplicate (i.e., three technical replicates of each biological replicate), along with a set of standards specific to the gene being analyzed. Each RNA preparation (the RNA used to create the cDNA) was also checked for DNA contamination by using crude RNA as the template and determining if amplification occurred. Relative expression was calculated as Mean Normalized Expression (MNE) [39].

### 2.10. Sequencing and Bioinformatic Analysis

Sanger sequencing was performed by the Micromon DNA sequencing facility at Monash University, Melbourne, Australia. Sequence data were analyzed using Chromas (Technelysium, South Brisbane, QLD, Australia) and databases on the National Centre for Biotechnology Information (NCBI) website. Homologs were identified using BLAST analysis. Protein sequences were aligned using MUSCLE [40] and visualized using Jalview [41]. Identification of putative binding motifs present in promoter regions was performed using PlantPAN 2.0 [42] and the Plant Transcription Factor Database [43]. Protein analysis was performed using ExPASy [44].

### 2.11. Subcellular Localization of Transiently Expressed SbGATA22

To determine the subcellular localization of the *Sb*GATA22 protein, transient expression of the *Sb*GATA22 protein N-terminal fused to GFP was assessed in *Nicotiana benthamiana* leaves. The plasmid containing the *SbGATA22* cDNA, identified in the Y1H screens, was used as the template for PCR amplification (Supplementary Table S2) with *Pfu* DNA polymerase (Promega, Madison, WI USA) and cloned into pENTR1A (Invitrogen, Waltham, MA, USA) [45]. The final construct was generated using Gateway technology (LR Clonase Life Technologies, Carlsbad, CA, USA) between pENTR1A:*Sb*GATA22 and the destination vector, pK7WGF2 containing the 35S promoter, and the enhanced green fluorescent protein (eGFP) [46]. pK7WGF2:*Sb*GATA22 was confirmed by sequencing to ensure in-frame fusion (primer details in Supplementary Table S2). pK7WGF2:*Sb*GATA22 was transformed into *Agrobacterium tumefaciens* electrocompetent AGL1 cells. The pK7WGF2 (vector only) was used as a control. The tomato bushy stunt virus (TBSV), p19, also in AGL1, was used to suppress post-transcriptional silencing [47]. Single colonies of each *Agrobacterium* culture were grown in 10 mL of liquid LB media (10 g L^−1^ Tryptone, 10 g L^−1^ NaCl, 5 g L^−1^ yeast extract, and pH7) with ampicillin (50 μg mL^−1^) or kanamycin (50 μg mL^−1^) for p19 and rifampicin (20 μg mL^−1^) overnight at 28 °C with shaking (180 rpm). *Agrobacterium* was pelleted via centrifugation at 2000× *g* for 10 min at room temperature and re-suspended to an OD_600_ = 0.5 in infiltration media (10 mM of MES (KOH pH 5.6), 10 mM of MgCl_2_, 0.5% glucose, and 200 μM of acetosyringone) and induced for 2–4 h with gentle agitation at room temperature. pK7WGF2:*Sb*GATA22 and pK7WGF2 *Agrobacterium* cultures were mixed 1:1 with p19 and Silwett L-77 (10 μL L^−1^) added prior to the infiltration of the *N. benthamiana* leaves. Plants were grown under lights for 3 days at 20 °C. GFP fluorescence was observed using a Zeiss Axioskop2 mot plus microscope (Zeiss, Oberkochen, Germany) using filter set 13 for GFP (excitation BP, 470/20; beam splitter FT, 495; emission BP, 505–530). Images were collected using the AxioVision software (AxioVs40 V 4.8.2.0).

### 2.12. Statistics

The statistics package Sigmaplot v 13 (Systat Software) was used for statistical analyses via one- or two-way ANOVA. For all tests, a *p*-value of <0.05 was considered significant. Means that were significantly different were compared post hoc using Tukey’s test. Data were log-transformed if required to satisfy the assumptions of normality.

## 3. Results

### 3.1. Identification of a Novel GATA Transcription Factor That Binds to the SbCYP79A1 Promoter

**Figure 1:**
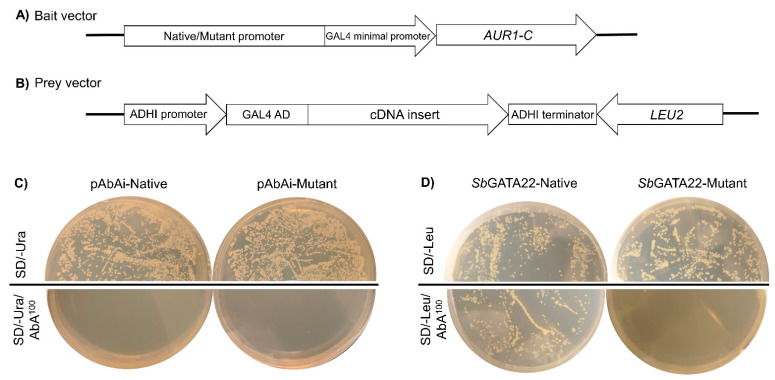
Yeast one-hybrid screening for regulatory proteins that bind to the *SbCYP79A1* (*Sb01g001200*) promoter region. Schematic diagrams of the (**A**) native/mutant bait vectors and (**B**) prey vectors containing inserts from the *Sorghum bicolor* (L.) Moench cDNA libraries. (**C**) The minimal inhibitory concentration of the antibiotic Aureobasidin A (AbA) required for cDNA library screening was determined by growing the bait yeast strains (Y1H pAbAi-Native and Y1H pAbAi-Mutant) on SD/-Ura media with or without the antibiotic. Images show that 100 ng mL^−1^ of AbA completely suppressed the growth of both bait strains and was, therefore, the appropriate inhibitory concentration to proceed with. (**D**) Y1H screening identified the transcription factor *Sb*GATA22 that interacts with the native *SbCYP79A1* promoter region. This is indicated by the growth of the Y1H *Sb*GATA22-Native strain on SD/-Leu/AbA^100^ media, as *Sb*GATA22 binds to the *SbCYP79A1* promoter region and drives the expression of the *AUR1-C* reporter gene, allowing for growth in the presence of the antibiotic AbA. When the putative GATA transcription factor binding motifs are mutated, *Sb*GATA22 does not bind, as indicated by a complete lack of growth of the Y1H *Sb*GATA22-Mutant strain on SD/-Leu/AbA^100^ media.

The *SbCYP79A1* native promoter bait fragment (1204 bp) was cloned into pAbAi vectors upstream of the *AUR1-C* reporter gene (Figure 1A) and introduced into the *S. cerevisiae* strain Y1H Gold. The resultant bait strain, Y1H pAbAi-Native, grew on SD/-Ura media (Figure 1C), indicating that the reporter sequence had stably integrated into the genome of the Y1H Gold yeast strain, which was confirmed by colony PCR. To determine whether endogenous yeast proteins recognized the bait sequence, the Y1H pAbAi-Native strain was streaked onto SD/-Ura media containing either 100, 150, or 200 ng mL^−1^ of the antibiotic Aureobasidin A (AbA). The growth of Y1H pAbAi-Native was completely suppressed by a minimum concentration of 100 ng mL^−1^ of AbA (Figure 1C).

Two cDNA libraries from *Sorghum bicolor* wild-type line BTx623 were constructed and screened: Screen 1—seeds, radicles, and emerging coleoptiles of seeds that had been imbibing for 24 and 48 h; Screen 2—coleoptile tips of seedlings 3 days post-germination (dpg). To ensure the adequate coverage of a cDNA library, it is essential to screen a minimum of 1 million clones. In this study, Screen 1 and Screen 2 resulted in the screening of 1.6 million and 3.3 million clones, respectively. Colonies over 2 mm in diameter after 5 days of incubation were re-streaked onto fresh SD/-Leu/AbA^100^ media, and any healthy colonies that appeared after 3 days were treated as positive clones, as growth suggested a positive interaction between the prey protein coded for by the cDNA insert and the *SbCYP79A1* promoter bait fragment (Figure 1B). Colony PCR was used to amplify cDNA inserts ranging in size from approximately 230 bp to 1400 bp, which were then sequenced. Several candidate transcription factors were identified; the insert coding for a GATA transcription factor 22 protein was identified in both Screen 1 and Screen 2 and was selected for further characterization. The open reading frame (ORF) of the protein, hereafter named *Sb*GATA22 based on NCBI nomenclature (also called *Sb*GATA32 by Yao et al. [48]), was found to be fused in-frame to the GAL4 activation domain. 

The cDNA insert of *Sb*GATA22 from Screen 1 is a full-length insert, 1435 bp long, which includes 41 bp of the 5′ untranslated region and 234 bp of the 3′ untranslated region including the polyA tail (Supplementary Figure S2). The ORF encodes a 386 amino acid polypeptide. The protein, *Sb*GATA22, is an uncharacterized long B-GATA with a conserved type IV zinc finger DNA-binding domain [49]. The polypeptide contains a run of nine histidines at the N-terminal region, a putative bipartite nuclear localization signal, and a conserved leucine–leucine–methionine (LLM) domain at the C-terminal region [50]. In plants, GATA transcription factors preferentially bind to the motif 5′ GATC 3′, with the core AT more conserved than the flanking sequence [51]. The *SbCYP79A1* promoter region analyzed has 22 putative GATA binding sites on the forward strand and 11 on the reverse (Supplementary Figure S3).

### 3.2. Confirmation of SbGATA22 Binding to GATA Transcription Factor Binding Motifs

To confirm that *Sb*GATA22 recognizes the putative GATA transcription factor binding motifs present in the *SbCYP79A*1 promoter region, a bait strain containing a mutated promoter fragment with all putative core GATA transcription binding sites changed from GAT to GTA on both the forward and reverse strands (Supplementary Figure S1) was produced (Y1H pAbAi-Mutant) and tested for autoactivation. As with the Y1H pAbAi-Native strain, 100 ng mL^−1^ of AbA suppressed the growth of Y1H pAbAi-Mutant strains on SD/-Ura media, confirming that endogenous yeast transcription factors were not binding to the mutant bait sequence (Figure 1C).

After the Y1H pAbAi-Mutant strain was transformed with the prey vector containing the *SbGATA22* insert, any interaction between the mutated *SbCYP79A1* promoter sequence and *Sb*GATA22 was analyzed in comparison with the native promoter sequence. The Y1H Native-*Sb*GATA22 strain (containing the native *SbCYP79A1* promoter–bait region and expressing the *Sb*GATA22 protein fused to a GAL4 activation domain) grew on both SD/-Leu media and SD/-Leu/AbA^100^ media, while the *Sb*GATA22-Mutant strain was unable to grow in the presence of 100 ng mL^−1^ of AbA (Figure 1D). This confirmed that *Sb*GATA22 binds to the core motif of GAT while being unable to recognize and bind to the mutated motif GTA. The interaction between the “prey” and “bait” in vivo is, therefore, deemed genuine.

### 3.3. Homology of SbGATA22

The *Sb*GATA22 protein sequence is homologous to putative GATA transcription factors in both C_3_ and C_4_ grasses, including maize (*Zea mays*), barley (*Hordeum vulgare*), and rice (*Oryza sativa*) (Supplementary Figure S4). The type IV zinc finger binding domain and the LLM domain are highly conserved across these species. *Sb*GATA22 has less homology to dicot plants, with 59% sequence identity (query coverage, 24%) to *Arabidopsis At*GATA21 (accession NP_200497.1) and even lower sequence similarity to GATA proteins in the dicotyledonous cyanogenic species cassava (*Manihot esculenta* Crantz) and *Lotus japonicus*.

To investigate the potential signaling pathways in which *Sb*GATA22 is involved, a 1 kb region upstream of the *SbGATA22* transcription start site (TSS) was analyzed for the presence of putative regulatory protein binding motifs (Supplementary Table S3). The putative binding motifs were predicted to be recognized by transcription factors involved in drought, light, and defense pathways, as well as phytohormone signaling pathways including auxin, ethylene, and gibberellic acid.

### 3.4. Subcellular Localization of SbGATA22

**Figure 2:**
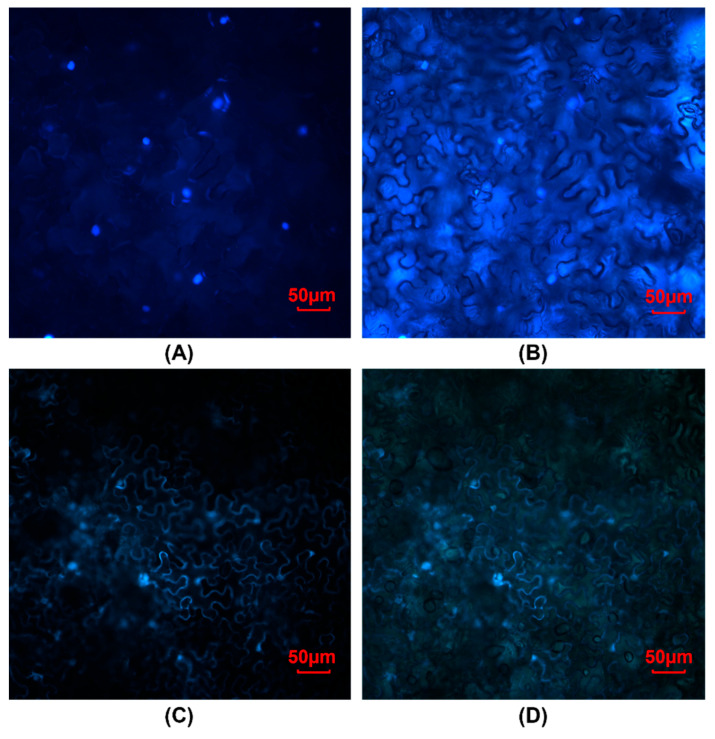
Localization of *Sb*GATA22 to the nucleus based on transient expression in leaves of *Nicotiana benthamiana*. (**A**) GFP fluorescence observed in *N. benthamiana* leaf cells transiently expressing the pK7WGF2:eGFP:*Sb*GATA22 construct demonstrating nuclear localization. (**B**) Identical view to (**A**) under both fluorescent and bright light to show cell structures. (**C**) GFP fluorescence observed in *N. benthamiana* leaf cells transiently expressing the pK7WGF2:eGFP vector only and (**D**) identical view to (**C**) under bright light and fluorescence to show cell structures.

Bioinformatic analysis of the protein sequence predicts that *Sb*GATA22 contains a bipartite nuclear localization signal. To determine if the protein is localized to the nucleus, N-terminal fusion constructs were generated with the enhanced green fluorescent protein (eGFP) and *Sb*GATA22 (pK7WGF2:eGFP:*Sb*GATA22). Transient expression in *N. benthamiana* confirms that *Sb*GATA22 is specifically localized to the nucleus (Figure 2A,B). The vector-only control, the pK7WGF2:eGFP construct, showed expression in the cytoplasm (Figure 2C,D).

### 3.5. Developmental Expression of SbGATA22

**Figure 3:**
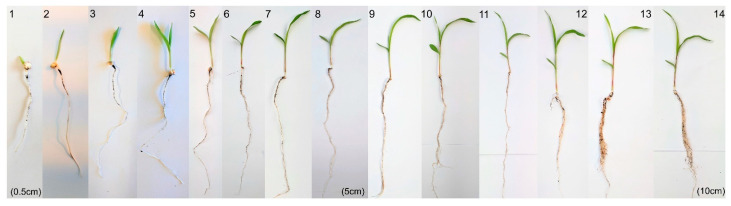
Representative examples showing growth of wild-type *Sorghum bicolor* (L.) Moench line BTx623 from 1 to 14 days post-germination. The height of the shoot was measured from the base of the shoot to the top ligule and is indicated at 1, 8, and 14 dpg.

**Figure 4:**
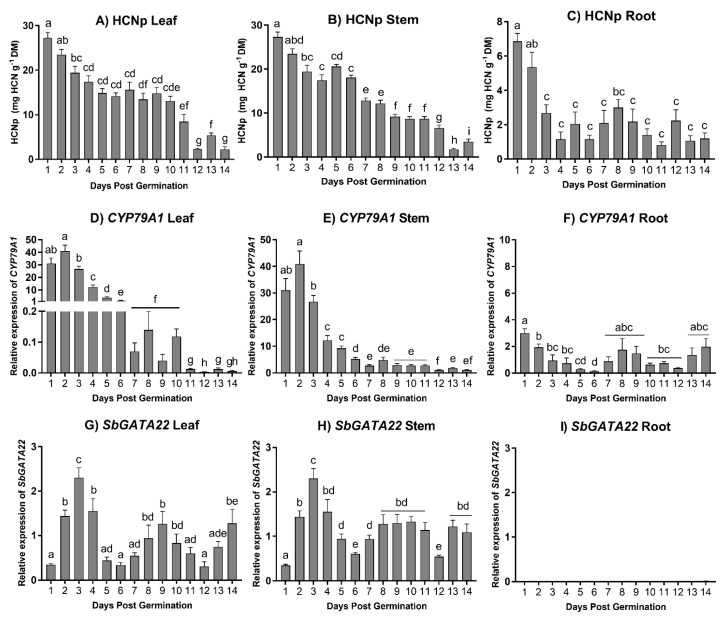
Hydrogen cyanide potential (mg HCN g^−1^ dry mass) and relative expression levels of *SbCYP79A1* and *SbGATA22* normalized to *Ubiquitin* in the leaf, stem, and root tissue of *Sorghum bicolor* wild-type line BTx623 from 1 day post-germination (dpg) to 14 dpg. Data denote mean ± SE (HCNp, *n* = 10; gene expression, *n* = 6, with 3 technical replicates for each biological replicate). Columns marked with identical letters are not significantly different at *p* < 0.05, analyzed using ANOVA and Tukey’s test.

As dhurrin concentrations are developmentally regulated in *S. bicolor* [1], the wild-type sorghum line BTx623 was grown and tissue-harvested from seedlings 1 to 14 days post-germination (dpg) (Figure 3), with the relative expression level of *SbGATA22* then analyzed and compared with *SbCYP79A1* expression and plant HCNp (Figure 4). The hydrogen cyanide potential of the sorghum leaf and stem tissue was highest following germination before immediately decreasing (Figure 4A,B). In the root tissue, HCNp was significantly lower than in the shoot tissue, though it was also highest directly following germination (Figure 4C). HCNp plateaued at 3 dpg in the roots, unlike in the shoot tissue, where dhurrin concentrations continued to decrease as the plants matured.

The relative expression pattern of *SbCYP79A1* corresponded closely to HCNp in all tissues (Figure 4D–F). In the leaf and stem tissue, *SbCYP79A1* expression was highest following germination, remaining high for the first three days before it rapidly decreased. This decrease was greatest in the leaf tissue, and by 7 dpg, the relative gene expression had decreased to below 0.2 from a peak of over 40. In the leaf and stem tissue, the relative expression of *SbGATA22* was low at 1 dpg before it increased and peaked at 3 dpg (Figure 4G,H). No expression of *SbGATA22* was observed in the root tissue (Figure 4I), consistent with data found in gene expression databases.

### 3.6. Expression of SbGATA22 in Response to Nitrogen Application

**Figure 5:**
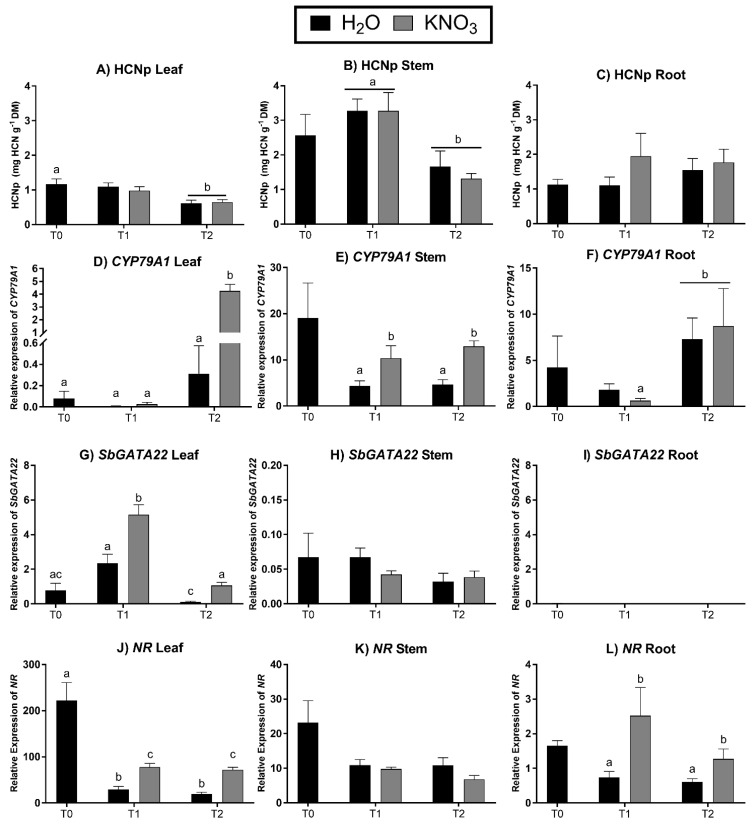
Hydrogen cyanide potential (mg HCN g^−1^ dry mass) and relative expression levels of *SbCYP79A1*, *SbGATA22*, and *nitrate reductase* (*NR*) normalized to *Ubiquitin* in the leaf, stem, and root tissue of *Sorghum bicolor* line BTx623 at 5-weeks old. A baseline harvest was performed (T0) before H_2_O or KNO_3_ was applied for either 2 days (T1) or 5 days (T2). Data denote mean ± SE (HCNp, *n* = 10; relative gene expression, *n* = 3, with 3 technical replicates for each biological replicate). Columns marked with identical letters are not significantly different at *p* < 0.05, analyzed using ANOVA and Tukey’s test.

As dhurrin concentrations can be affected by nitrogen application, and GATA transcription factors are involved in nitrogen metabolism [12,52,53], the expression of *SbGATA22* in response to KNO_3_ application was investigated. Hydrogen cyanide potential and the relative expression of *SbCYP79A1*, *SbGATA22*, and *nitrate reductase* (*NR)* were analyzed in 5-week-old plants (T0) and following the application of either H_2_O or 25 mM KNO_3_ for 2 days (T1) and 5 days (T2). No induction of HCNp was observed in any tissue following KNO_3_ application (Figure 5A–C). Despite this, the relative expression of *SbCYP79A1* increased in response to KNO_3_ in the leaf tissue at T2 and the stem tissue at T1 and T2 (Figure 5D–F). *SbGATA22* expression was higher in the leaf tissue of plants treated with KNO_3_ compared with control plants at T1 and T2, though no induction occurred in the stem or root tissues (Figure 5G–I). The relative expression of *nitrate reductase* increased in the leaf and root tissues at T1 and T2 in response to KNO_3_ (Figure 5J,L).

## 4. Discussion

In this study, the developmental regulation of the dhurrin biosynthetic genes in sorghum was investigated. This was achieved by screening cDNA libraries made from mRNA that was extracted during the early stages of plant development. In sorghum, dhurrin concentrations peak at three to four days post-germination, with transcript levels of the cytochrome P450 genes involved in dhurrin biosynthesis (*SbCYP79A1* and *SbCYP71E1*) highest in the short period following germination [8,20]. Thus, we aimed to identify regulatory proteins present in sorghum during the stages of maximum *SbCYP79A1* expression and dhurrin production. Screen 1 pooled seeds that had been imbibing for 24 h (emerging radicle) and 48 h (radicle and emerging coleoptile); Screen 2 used the tips of coleoptiles 3 days post-germination, this being the tissue and time-point where dhurrin concentration is at a maximum [1]. As important cis-regulatory elements are not known, a native promoter fragment containing 1204 bp of the *SbCYP79A1* promoter region was used as bait. While many Y1H screens use trimers of core binding motifs as the bait sequence, the use of large promoter fragments has also been successful, and their use may result in a broader range of transcription factors being identified [34,54]. Over 5 million clones were screened in this study, with the transcription factor *Sb*GATA22 found to bind to the *SbCYP79A1* promoter region at both developmental stages analyzed and, therefore, selected for further analysis.

### 4.1. GATA Transcription Factors in Plant Development and Stress Responses

GATA transcription factors are found in all eukaryotes and are characterized by a type IV zinc finger binding domain [50,52,53]. In plants, GATA transcription factors preferentially bind to GATC motifs, with the core AT sequence most highly conserved [51,55]. We found that *Sb*GATA22 recognizes GAT motifs, as the mutation of these sites into GTA resulted in no expression of the *AUR1-C* reporter gene (Figure 1D).

GATA binding motifs were first found to be enriched in promoter regions of circadian clock and light-regulated genes [56], and in *Arabidopsis*, they have been shown to be involved in nitrogen signaling, germination control, greening, senescence, and flower development [49,57]. GATAs are also involved in the environmental regulation of specialized metabolites and have recently been found to activate the promoters of five light-responsive genes in the biosynthetic pathway of the terpenoid indole alkaloid vindoline in the periwinkle *Catharanthus roseus* [58].

*Sb*GATA22 is classified as a long B-GATA with a C-terminal leucine–leucine–methionine (LLM) domain [50]. There are 11 B-GATA transcription factors in *Arabidopsis*; the first two characterized were the paralogous GATA NITRATE-INDUCIBLE, CARBON METABOLISM-INVOLVED (GNC), and CYTOKININ-RESPONSIVE GATA FACTOR 1/GNC-LIKE (CGA1/GNL) [57,59]. In *Arabidopsis*, GNC and CGAI/GNL are involved in light signaling pathways and regulated by nitrogen availability and the phytohormones gibberellic acid, auxin, and cytokinin [52,53,60]. They can act as either activators or repressors of downstream target genes. *Sb*GATA22 is homologous to GNC (*At*GATA21), and while GNC is well characterized in *Arabidopsis*, the same cannot be said for monocot homologs.

In monocots, GATA transcription factors have predominantly been characterized in rice (*Oryza sativa*), where they are involved in similar pathways to *Arabidopsis* [61]. In rice, GATA transcription factors have also been implicated in abiotic stress responses, with gene expression changing in response to salinity, drought, and abscisic acid (a phytohormone with roles in drought and salt stress) [62]. Despite the knowledge of GATA transcription factor function in rice, the extent of functional conservation between monocots has only recently started to be explored and remains largely computational [48]. These computational analyses in other monocot species, such as wheat [63] and foxtail millet [64], also predict roles for GATA transcription factors in response to stress and in hormone signaling pathways. While GATA sequences tend to be more highly conserved among monocots than to more distantly related dicot species [48,63,65], functional characterization is needed to understand their roles in specific pathways and responses.

In this study, *SbGATA22* was expressed in the shoots at all developmental stages analyzed, including directly following germination (Figure 4). Interestingly, there was no expression of *SbGATA22* in the root tissue, consistent with other studies that indicate that dhurrin expression is independently regulated in roots and shoots [12,66]. A lack of GATA transcription factor expression in root tissues has also been observed in other species and may be related to their role in greening [57,67]. However, as the seedlings used for RNA extraction in the Y1H screens were etiolated and harvested in the dark, light is not required for *SbGATA22* expression, which is evidence that, in sorghum, *Sb*GATA22 is likely involved in pathways other than greening. This is further supported by the presence of cis-regulatory elements in the *SbGATA22* promoter region that are not only involved in light signaling pathways but also in defense, carbon metabolism, and drought stress (Supplementary Table S3). Notably, in cyanogenic species, CNglcs are regulated in response to light, drought, and nitrogen availability [1,68], the same pathways in which GATA transcription factors are involved.

The transcription profile of *Sb*GATA22 may suggest that it is a transcriptional repressor of *SbCYP79A1*, as shown by the leaf and stem tissue expression of *SbGATA22* increasing when *SbCYP79A1* was at a maximum (1–3 dpg) and inversely correlating with *SbCYP79A1* expression during plant development (Figure 4). However, this cannot be definitively stated, as *Sb*GATA22 is likely involved in other pathways and, due to this, expression relative to *SbCYP79A1* may differ. Furthermore, since *SbCYP79A1* is expressed in the roots, when *SbGATA22* expression is absent, it can be deduced that *Sb*GATA22 is unlikely to be the sole regulator of *SbCYP79A1* expression in sorghum. Interestingly, CGAI/GNL in rice has reduced expression during dark periods [67], which is also when CNglcs have been found to increase in concentration in another well-studied cyanogenic species, cassava [68]. If GATA transcription factors are involved in CNglc regulation, their reduced expression during dark periods may also correspond to them acting as repressors of CNglc biosynthetic genes.

In animals, GATA transcription factors have binding partners known as FRIENDS OF GATA (FOGs) [69,70]. These transcriptional co-factors interact with GATA proteins via their own zinc finger domains and either enhance or repress GATA activity. No genes homologous to FOGs have been found in plant genomes. However, yeast two-hybrid screens have identified some binding partners of plant GATAs, including the transcriptional repressors SIN3-Like1, involved in the histone deacetylase complex, and TOPLESS, involved in jasmonate, auxin, and defense responses [71,72]. Though the precise consequences of these interactions are not known, this suggests that the functions of GATA transcription factors in plants are also modulated by co-regulators.

### 4.2. SbGATA22 Expression in Response to Nitrogen Application

As GATA transcription factors are linked to nitrogen signaling, KNO_3_ was applied to 5-week-old sorghum plants for either two (Time 1) or five (Time 2) days. This followed the protocol of Busk and Møller [20], where a large induction in dhurrin concentration in leaves and stems was observed following KNO_3_ application. Their study also confirmed that the induction in dhurrin was due to NO_3_^−^ and not potassium. Here, dhurrin was not induced at either harvest (Figure 5A–C). However, increased *nitrate reductase* (*NR*) expression indicated that the plants did respond to the nitrate application. *NR* expression was higher in both leaf and root tissues of the treatment group compared with the controls at both T1 and T2 (Figure 5J–L). Leaf *SbCYP79A1* expression was over ten-fold higher in the treatment group than the control at Time 2, while in the stem, it was twice as high in the treatment group at Time 1 and Time 2. As increased *SbCYP79A1* expression did not result in higher dhurrin concentrations in either the leaves or stem, it is possible that dhurrin production was induced but turned over at an equal rate via the endogenous turnover pathway rather than accumulated [6,7]. The expression of *SbGATA22* was also higher in the leaf tissue when nitrate was applied at both Time 1 and Time 2, but no changes in expression were seen in the stem or the root (Figure 5G–I). As with the developmental expression data, *Sb*GATA22 fits the profile of a repressor more than that of an activator as, once again, high *SbGATA22* expression correlated to lower *SbCYP79A1* expression.

## 5. Conclusions

To date, attempts to alter the cyanogenic status of plants have predominantly focused on knocking out CNglc biosynthetic genes to produce acyanogenic individuals, which can affect plant growth rates and stress tolerance. Research into the molecular regulation of CNglc biosynthesis provides new targets for the manipulation of cyanogenic potential and may enable a reduction in (rather than the removal of) CNglcs in cyanogenic species. The ability to alter the induction of CNglc biosynthetic genes in response to environmental factors would also reduce the risks of toxicity when used for food and forage. This study is the first to identify candidate regulators of sorghum’s key dhurrin biosynthetic gene, *SbCYP79A1*. We have established a preliminary point for investigation into the transcription factors regulating CNglc production in sorghum. Further characterization of *Sb*GATA22 will reveal whether it has a direct role in controlling *SbCYP79A1* expression in planta or if additional co-factors are required.

## Data Availability

Data is contained within the article.

## Supplementary Materials

The following are available online at https://www.mdpi.com/article/10.3390/life14040470/s1: Figure S1: The sequence of the native and mutant promoter “bait” fragments of *SbCYP79A1* (*Sb01g001200*) cloned into pUC57 and then into pAbAi. Figure S2: Nucleotide and protein sequence of *Sb*GATA22. Figure S3: Core GATA transcription-binding motifs present on both forward and reverse strands of the *SbCYP79A1* promoter region. Figure S4: Alignment of the *Sb*GATA22 amino acid sequence with its closest homologs. Table S1: Primers used in qPCR. Table S2: Primers used in cloning and sequencing *SbGATA22* for nuclear localization. Table S3: Putative transcription factor binding motifs located in the 1000 bp region upstream of the *SbGATA22* transcription start site.
