# Supplementary material for: The Putative GATA Transcription Factor SbGATA22 as a Novel Regulator of Dhurrin Biosynthesis
Source: Life (Basel). 2024 Apr 3;14(4):470. doi: 10.3390/life14040470 (PMC11051066; doi:10.3390/life14040470)
Supplement: Supplementary file 1 [file life-14-00470-s001.zip › life-2879338-supplementary.pdf]

Supplementary Material

The putative GATA transcription factor *Sb*GATA22 as a novel regulator of dhurrin biosynthesis

Viviana C. Rosati, Alicia A. Quinn, Roslyn M. Gleadow and Cecilia K. Blomstedt

Supplementary Figures

Fig S1: Sequence of the native and mutant promoter "bait" fragments of *SbCYP79A1* (*Sb01g001200*) cloned into pUC57 and then into pAbAi.

Fig S2: Amino acid sequence of *Sb*GATA22 aligned to the nucleotide insert sequence from Screen 1.

Fig S3: Core GATA transcription binding motifs present on both forward and reverse strands of the *SbCYP79A1* promoter region.

Fig S4: Alignment of the *Sb*GATA22 (accession XP_002437185.1) amino acid sequence with its closest homologues.

Supplementary Tables

Table S1: Primers used in qPCR

Table S2: Primers used in cloning *SbGATA22* into pK7WGF2 for nuclear localisation.

Table S3: Putative transcription factor binding motifs located in the 1000bp region upstream of the *SbGATA22* transcription start site (TSS).

```
Native      GCATACCGATCGATGCGAAAAAAAAACCCAGGAGACTCTCGAAGATCTACAGATTAAATC    60
Mutant      GCATACCGTACGTAGCGAAAAAAAAACCCAGGAGACTCTCGAAGTACTACAGTATAAGCT    60
            ********  **  ******************************  ******  ***

Native      TAAGTTTCCACGGTCCAATATGCCGATTGGTAAGTTAGAACGAAGTAGGGAGAGAGAAAA    120
Mutant      TAAGTTTCCACGGTCCAATATGCCGTATGGTAAGTTAGAACGAAGTAGGGAGAGAGAAAA    120
            *************************  *********************************

Native      AAAACCATAGGGAGGAGGAAGACAAGAAAAAAGACTCGGGCAGGAAGAGTGTATGCTGGT    180
Mutant      AAAACCATAGGGAGGAGGAAGACAAGAAAAAAGACTCGGGCAGGAAGAGTGTATGCTGGT    180
            ************************************************************

Native      TTAAAATAGGAGCTCGGTGTCTTAGATCATGGTGCCAAATTTGACGCCAATGGTAATCGC    240
Mutant      TTAAAATAGGAGCTCGGTGTCTTAGTACATGGTGCCAAATTTGACGCCAATGGTAGCTGC    240
            *************************  ****************************   **

Native      GTCGAGGTCTCTGCCACGTCAACGCCATGTCTGCGTCAACACCAACATGGCACAACGACC    300
Mutant      GTCGAGGTCTCTGCCACGTCAACGCCATGTCTGCGTCAACACCAACATGGCACAACGACC    300
            ************************************************************

Native      TTGGCGCCTAATTGGGCTGGCGCCGAGATGTGTGACCTTGGCGGCAATTACAACGGCGCT    360
Mutant      TTGGCGCCTAATTGGGCTGGCGCCGAGTAGTGTGACCTTGGCGGCAATTACAACGGCGCT    360
            ***************************  *******************************

Native      GAGCAGAGGGTCTAGATTTTGAAAGCATACCTACCGGGACAGATTTATGAAAATCTTTTA    420
Mutant      GAGCAGAGGGTCTAGTATTTGAAAGCATACCTACCGGGACAGTATTATGAAAGCTTTTTA    420
            ***************  *************************  ********   *****

Native      AAAAAAAGAAGCAAAAAAACAAAAAAAAATGCCAGACATGCGGTGGCTCTTTGGCACGCC    480
Mutant      AAAAAAAGAAGCAAAAAAACAAAAAAAAATGCCAGACATGCGGTGGCTCTTTGGCACGCC    480
            ************************************************************

Native      TGCGTGACACCCCTCCCTTCATGCACGCCGACAAGCAGACAGAAAAAGCCTTGGTGAGAG    540
Mutant      TGCGTGACACCCCTCCCTTCATGCACGCCGACAAGCAGACAGAAAAAGCCTTGGTGAGAG    540
            ************************************************************

Native      AAAGGGGATGACATGGGCCCACATGTATGAGTGAGGGAGAGGGCAGTCGTTGCGGTATTT    600
Mutant      AAAGGGGTAGACATGGGCCCACATGTATGAGTGAGGGAGAGGGCAGTCGTTGCGGTATTT    600
            *******  ***************************************************

Native      TGGGTAATGTGAAAATACAACAATCTGGTTCCCATCTTGTTTCAAAGGTAGAAAATGGCA    660
Mutant      TGGGTAATGTGAAAATACAACAGCTTGGTTCCCGCTTTGTTTCAAAGGTAGAAAATGGCA    660
            **********************   ********   ************************

Native      TGGATCAAGTATAGAAACAATTGTATTGGCAAGTTTCCAAATACATGAATAGTAATAAGT    720
Mutant      TGGTACAAGTATAGAAACAATTGTATTGGCAAGTTTCCAAATACATGAATAGTAATAAGT    720
            ***  *******************************************************

Native      GTGAAAATTATAATGGCATGGTTCAAATAAACCCCAAAAATATTGTTAGTATTGTAATAA    780
Mutant      GTGAAAATTATAATGGCATGGTTCAAATAAACCCCAAAAATATTGTTAGTATTGTAATAA    780
            ************************************************************

Native      TAAAGAAAACCAAAACGGTTCGGACTGGGCTTGTCCCCTCAACACTATCGTCATGGGTAG    840
Mutant      TAAAGAAAACCAAAACGGTTCGGACTGGGCTTGTCCCCTCAACACTGCTGTCATGGGTAG    840
            **********************************************   ***********

Native      CGCACACAGCATGATGTGCTCGATCATTCTGAGCTCCCACATCATGTGCATGCTGCATGG    900
Mutant      CGCACACAGCATGTAGTGCTCGTACATTCTGAGCTCCCACGCTATGTGCATGCTGCATGG    900
            *************  *******  ****************   *****************

Native      TCGATGCGCTGCACACAGTTAGCTGTGGGACTGTGCATGCACGCGTGGTTGGTGGCGATC    960
Mutant      TCGTAGCGCTGCACACAGTTAGCTGTGGGACTGTGCATGCACGCGTGGTTGGTGGCGTAC    960
            ***  ****************************************************  *

Native      CAGCATCCCATAAATACCTGCGAACCGCCGTGTGTTCTGCCTCAAGCAGGAGCACATACA    1020
Mutant      CAGCGCTCCATAAATACCTGCGAACCGCCGTGTGTTCTGCCTCAAGCAGGAGCACATACA    1020
            ****   *****************************************************
```

```
Native      GCTAGCTAGCTCATCGGGTGATCGATCAGTGAGCTCTCTCTTTGGCCTAGCTAGCTGCTA     1080
Mutant      GCTAGCTAGCTCGCTGGGTGTACGTACAGTGAGCTCTCTCTTTGGCCTAGCTAGCTGCTA     1080
            ************   *****  **  **********************************

Native      GCAGTGCAGGTAGCCAATCAAAGCAGAAGAACTCGATCGATCGATCATCACGATCGCTGC     1140
Mutant      GCAGTGCAGGTAGCCAGCTAAAGCAGAAGAACTCGTACGTACGTACGCTACGTACGCTGC     1140
            ****************   ****************  **  **  *   ***  ******

Native      TAGCTAGCTAGCTGCTCGCTCTCACACTAGCTACGTGTTTTTGTTAATTTGATATATATA     1200
Mutant      TAGCTAGCTAGCTGCTCGCTCTCACACTAGCTACGTGTTTTTGTTAATTTGTAATATATA     1200
            ***************************************************  *******
```

Supplementary Figure S1: Native and mutant promoter “bait” fragments of *SbCYP79A1* (*Sb01g001200*) cloned into pUC57 and then into pAbAi. The mutated promoter fragment had all putative core GATA transcription binding sites changed from GAT to GTA on both the forward and reverse strands. Putative binding sites were identified using PlantPAN 2.0 (Chow et al., 2015).

M S A I Y M

1 *GGCGCAAGCAAATTCCTGATCTCATCAGTCCCTATAGCTCCC***ATG**TCTGCCATCTACATG

S Q L S T A L P L M E G D H H H H H H

61 AGCCAGCTCTCCACTGCTCTCCCTCTCATGGAGGGAGACCACCACCACCACCACCACCAC

H H Q G H F Q A F T L P K D P P I L F P

121 CACCACCAAGGCCACTTCCAAGCCTTCACGCTGCCAAAGGATCCCCCAATCCTTTTCCCC

F V I S N S S A S E S S L S Y G S A D H

181 TTTGTGATCAGCAATAGTAGCGCCAGCGAAAGCAGTCTGAGCTATGGATCGGCAGATCAT

H L L R Q H R Q T M L E P Q H M I G G S

241 CACTTGTTGAGGCAGCATCGTCAAACTATGCTCGAGCCCCAACATATGATTGGTGGATCG

S S A A S S V F A T P F P T V E S I R D

301 TCATCAGCTGCGAGTAGTGTCTTTGCGACGCCGTTCCCGACTGTGGAGAGCATCCGTGAC

D M I E P A S Y D P Y D M G K L H Q V V

361 GACATGATCGAGCCTGCCTCGTACGATCCATACGATATGGGGAAGCTGCACCAGGTGGTC

G G G S S M D A C S W T P P A A K M R I

421 GGTGGCGGGTCGTCGATGGATGCTTGCAGCTGGACGCCGCCGGCGGCCAAGATGAGGATC

T R K A T A A D P S G A G K K P R R R A

481 ACGAGGAAGGCCACTGCCGCCGATCCCAGTGGTGCCGGGAAGAAGCCGAGGAGAAGGGCG

H Q A A G Y D A D I N M S G Q P N L G V

541 CATCAGGCAGCAGGGTACGACGCCGACATCAACATGAGCGGCCAACCAAATTTGGGTGTT

I R V C S D C N T T K T P L W R S G P C

601 ATTAGGGTGTGCTCCGACTGCAACACCACCAAGACGCCCTTGTGGAGGAGTGGTCCTTGC

G P K S L C N A C G I R Q R K A R R A M

661 GGCCCCAAGTCGCTCTGCAACGCGTGCGGTATCAGGCAGCGGAAGGCACGGCGGGCGATG

M A A A S T S G P A A V P A T D S D K A

721 ATGGCCGCCGCGTCTACCTCCGGGCCAGCAGCAGTGCCCGCCACCGACAGCGATAAGGCC

S P S N A A G A A A A H P K V K K E K R

781 TCGCCGAGCAACGCCGCCGGGGCCGCAGCAGCACACCCCAAGGTGAAGAAGGAGAAGAGA

S V D V D R S L P F K K R C K V V Q V Q

841 TCAGTGGACGTGGACCGGTCGCTGCCGTTCAAGAAACGGTGCAAGGTGGTCCAGGTCCAG

Q D H A A A V A A P A A A T D R P A V V

901 CAGGATCACGCTGCGGCCGTCGCAGCTCCTGCCGCGGCCACTGACAGGCCGGCTGTCGTC

V Q Q A A T A A E V G D D D A C P S R D

961 GTGCAGCAGGCGGCCACCGCCGCCGAGGTTGGTGACGACGACGCCTGTCCGAGCAGGGAC

L L V D D I G G L I S W S R S P P A A P

1021 CTGCTTGTCGACGACATTGGTGGGCTCATCAGCTGGAGCAGGAGTCCGCCGGCGGCTCCC

A S A D A A S C S F R A S P A L P V Q Q

1081 GCATCTGCAGATGCTGCCTCCTGCAGTTTCCGGGCGTCGCCGGCGTTGCCGGTGCAGCAG

D E I T D A A M L L M T L S C G L V R S

1141 GACGAGATCACGGACGCTGCCATGCTGCTCATGACGCTGTCCTGCGGGCTTGTCCGAAGC

*

1201 **T*GA***TCGACGATCGATCTACCTCTACAAAGGCCCCAGCACCATAGGCTTTTTGCAGCTAGC*

1261 *TACCTCTAGCTAGTGCCTTTGACTGTTAGTGTTACTGTTAGTGTTGTTACCATCGTCGTT*

1321 *TACCCTACGACCTGTAGTTTTTTTTATTTGGCTTCTTGTTCTTGTTGCCAGTGTAACCCG*

1381 *TGTCACTGTTCAAGTCATGTGATTCGTTTGAGAAAAAAAAAAAAAAAAAAAAAAA*

Supplementary Figure S2: Amino acid sequence of *Sb*GATA22 aligned to the nucleotide insert sequence from Screen 1. The nucleotide sequence is 1435bp long, which includes 41bp of the 5’ untranslated region and 234bp of the 3’ untranslated region including the polyA tail (*italics*). The open reading frame (ORF) encodes a 386 amino acid polypeptide, the putative stop codon is indicated (*). The polypeptide contains a conserved type IV zinc finger DNA-binding domain, a run of 9 histidines at the N-terminal region (underlined), a putative bipartite nuclear localization signal (dashed underline), and a conserved Leucine-Leucine-Methionine (LLM) domain at the C-terminal region (bold).

```
5′ GCATACCGATCGATGCGAAAAAAAAACCCAGGAGACTCTCGAAGATCTACAGATTAAATC      -1145
3′ CGTATGGCTAGCTACGCTTTTTTTTTGGGTCCTCTGAGAGCTTCTAGATGTCTAATTTAG

5′ TAAGTTTCCACGGTCCAATATGCCGATTGGTAAGTTAGAACGAAGTAGGGAGAGAGAAAA      -1085
3′ ATTCAAAGGTGCCAGGTTATACGGCTAACCATTCAATCTTGCTTCATCCCTCTCTCTTTT

5′ AAAACCATAGGGAGGAGGAAGACAAGAAAAAAGACTCGGGCAGGAAGAGTGTATGCTGGT      -1025
3′ TTTTGGTATCCCTCCTCCTTCTGTTCTTTTTTCTGAGCCCGTCCTTCTCACATACGACCA

5′ TTAAAATAGGAGCTCGGTGTCTTAGATCATGGTGCCAAATTTGACGCCAATGGTAATCGC      -965
3′ AATTTTATCCTCGAGCCACAGAATCTAGTACCACGGTTTAAACTGCGGTTACCATTAGCG

5′ GTCGAGGTCTCTGCCACGTCAACGCCATGTCTGCGTCAACACCAACATGGCACAACGACC      -905
3′ CAGCTCCAGAGACGGTGCAGTTGCGGTACAGACGCAGTTGTGGTTGTACCGTGTTGCTGG

5′ TTGGCGCCTAATTGGGCTGGCGCCGAGATGTGTGACCTTGGCGGCAATTACAACGGCGCT      -845
3′ AACCGCGGATTAACCCGACCGCGGCTCTACACACTGGAACCGCCGTTAATGTTGCCGCGA

5′ GAGCAGAGGGTCTAGATTTTGAAAGCATACCTACCGGGACAGATTTATGAAAATCTTTTA      -785
3′ CTCGTCTCCCAGATCTAAAACTTTCGTATGGATGGCCCTGTCTAAATACTTTTAGAAAAT

5′ AAAAAAAGAAGCAAAAAAACAAAAAAAAATGCCAGACATGCGGTGGCTCTTTGGCACGCC      -725
3′ TTTTTTTCTTCGTTTTTTTGTTTTTTTTTACGGTCTGTACGCCACCGAGAAACCGTGCGG

5′ TGCGTGACACCCCTCCCTTCATGCACGCCGACAAGCAGACAGAAAAAGCCTTGGTGAGAG      -665
3′ ACGCACTGTGGGGAGGGAAGTACGTGCGGCTGTTCGTCTGTCTTTTTCGGAACCACTCTC

5′ AAAGGGGATGACATGGGCCCACATGTATGAGTGAGGGAGAGGGCAGTCGTTGCGGTATTT      -605
3′ TTTCCCCTACTGTACCCGGGTGTACATACTCACTCCCTCTCCCGTCAGCAACGCCATAAA

5′ TGGGTAATGTGAAAATACAACAATCTGGTTCCCATCTTGTTTCAAAGGTAGAAAATGGCA      -545
3′ ACCCATTACACTTTTATGTTGTTAGACCAAGGGTAGAACAAAGTTTCCATCTTTTACCGT

5′ TGGATCAAGTATAGAAACAATTGTATTGGCAAGTTTCCAAATACATGAATAGTAATAAGT      -485
3′ ACCTAGTTCATATCTTTGTTAACATAACCGTTCAAAGGTTTATGTACTTATCATTATTCA

5′ GTGAAAATTATAATGGCATGGTTCAAATAAACCCCAAAAATATTGTTAGTATTGTAATAA      -425
3′ CACTTTTAATATTACCGTACCAAGTTTATTTGGGGTTTTTATAACAATCATAACATTATT

5′ TAAAGAAAACCAAAACGGTTCGGACTGGGCTTGTCCCCTCAACACTATCGTCATGGGTAG      -365
3′ ATTTCTTTTGGTTTTGCCAAGCCTGACCCGAACAGGGGAGTTGTGATAGCAGTACCCATC

5′ CGCACACAGCATGATGTGCTCGATCATTCTGAGCTCCCACATCATGTGCATGCTGCATGG      -305
3′ GCGTGTGTCGTACTACACGAGCTAGTAAGACTCGAGGGTGTAGTACACGTACGACGTACC

5′ TCGATGCGCTGCACACAGTTAGCTGTGGGACTGTGCATGCACGCGTGGTTGGTGGCGATC      -245
3′ AGCTACGCGACGTGTGTCAATCGACACCCTGACACGTACGTGCGCACCAACCACCGCTAG

5′ CAGCATCCCATAAATACCTGCGAACCGCCGTGTGTTCTGCCTCAAGCAGGAGCACATACA      -185
3′ GTCGTAGGGTATTTATGGACGCTTGGCGGCACACAAGACGGAGTTCGTCCTCGTGTATGT

5′ GCTAGCTAGCTCATCGGGTGATCGATCAGTGAGCTCTCTCTTTGGCCTAGCTAGCTGCTA      -125
3′ CGATCGATCGAGTAGCCCACTAGCTAGTCACTCGAGAGAGAAACCGGATCGATCGACGAT

5′ GCAGTGCAGGTAGCCAATCAAAGCAGAAGAACTCGATCGATCGATCATCACGATCGCTGC      -65
3′ CGTCACGTCCATCGGTTAGTTTCGTCTTCTTGAGCTAGCTAGCTAGTAGTGCTAGCGACG

5′ TAGCTAGCTAGCTGCTCGCTCTCACACTAGCTACGTGTTTTTGTTAATTTGATATATATA      -5
3′ ATCGATCGATCGACGAGCGAGAGTGTGATCGATGCACAAAAACAATTAAACTATATATAT

5′ TATA                                                                -1
3′ ATAT
```

Supplementary Figure S3: Core GATA transcription binding motifs: 5’ GAT 3’ present on both forward and reverse strands of the *SbCYP79A1* promoter region identified using PlantPAN 2.0 (Chow et al., 2015).

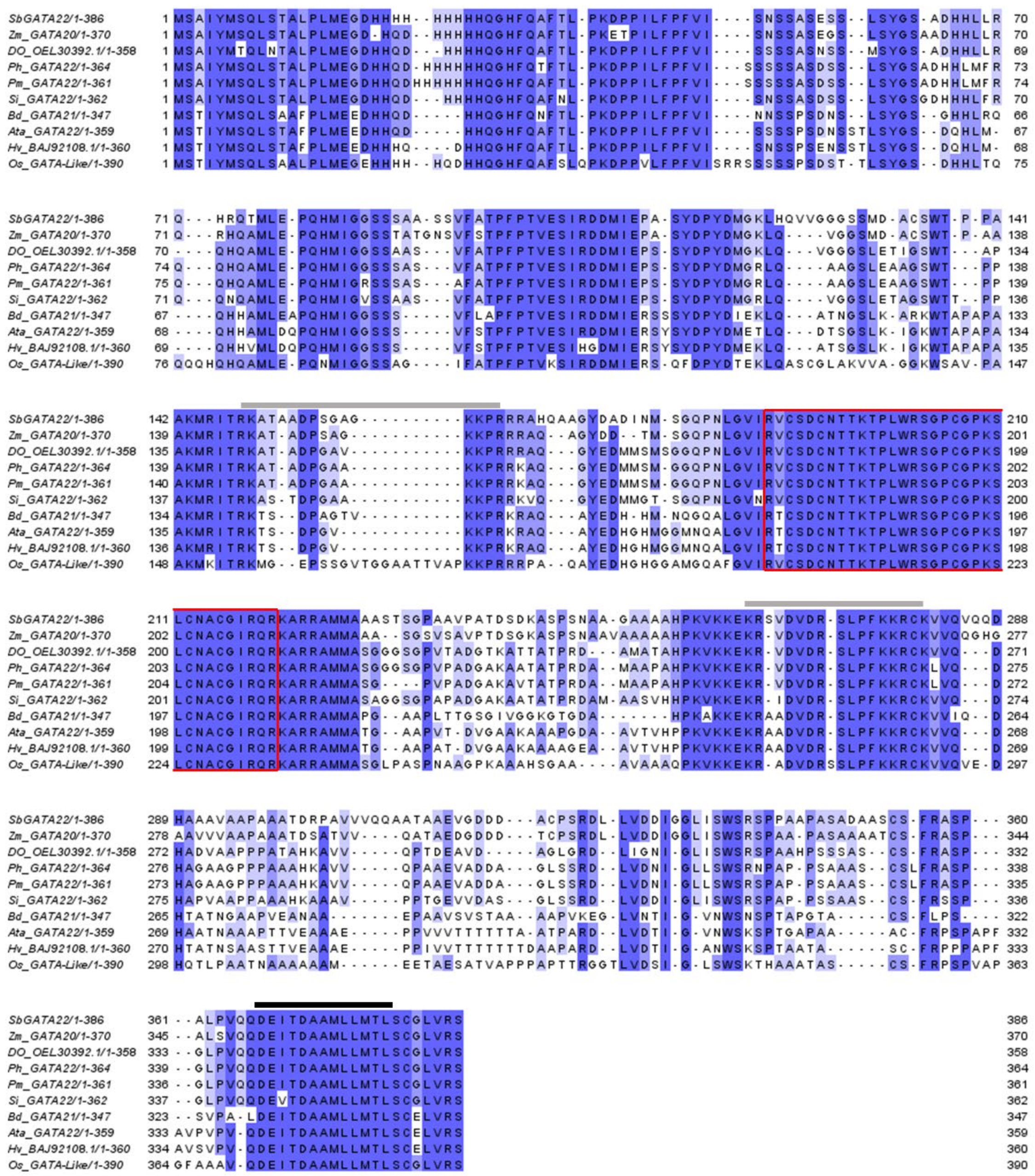

Supplementary Figure S4. Alignment of the *Sb*GATA22 (accession XP_002437185.1) amino acid sequence with its closest homologues from the following species *Zea mays* (*Zm*, accession: ACG27673.1); *Dichanthelium oligosanthes* (*Do*, accession OEL30392.1); *Panicum hallii* (*Ph*, accession XP_025812836.1); *Panicum miliaceum* (*Pm*, accession RLM56087.1); *Setaria italica* (*Si*, accession XP_004966043.1); *Brachypodium distachyon* (*Bd*, XP_003563721.1); *Aegilops tauschii* (*Ata*, accession XP_020170490.1); *Hordeum vulgare* (*Hv*, accession BAJ92108.1); *Oryza sativa* Japonica (*Os*, accession XP_015641328.1). The zinc finger binding domain (C-X$_2$-C-X$_{18}$-C-X$_2$-C) is indicated by a red box, the LLM-domain by a black line, and the putative bipartite nuclear localization signal by a gray line.

Supplementary Table S1: Primers used in qPCR

Primer Name	Primer Sequence (5′ – 3′)	Tm °C	Size (bp)
SbCYP79A1 F	CATGCTCTTCGGAAGGCTCCT	66	162
SbCYP79A1 R	ATGGAGATGGACGGGTAGAGGT		
SbGATA22 F	TGACGACGACGCCTGTCCGA	66	179
SbGATA22 R	TGAGCAGCATGGCAGCGTCC		
NR F	CCCATGTACCAGGTCATCCA	63	172
NR R	CCTGGTCGATCACGTACCAC		
Ubiquitin F	CGGAGGAGCTCTATGCCACA	66	122
Ubiquitin R	AAGACGCTCCACTGCAGCAT		

Supplementary Table S2: Primers used in cloning *SbGATA22* into pENTR1A vector and subsequent confirmation of the final destination vector, pK7WGF2, to ensure that *SBGATA22* is in the correct reading frame with GFP.

Primer Name	Primer Sequence 5′– 3′	Tm °C	Size (bp)
**Cloning**			
SbGATA22 F	TTAGCCGGTACCtaATAGCTCCCATGTCTGCC	64	1195
SbGATA22 R	AAGAATGCGGCCGCCGATCAGCTTCGGACAAG		
**Sequencing**			
pK7WGF2:GATA2+ F	TCACCACTTTGTACAAGAAAGCTGGG	62	
pK7WGF2:GATA2+ R	GCTGGAGTTCGTGACCGCC	63	

Forward primer contains an extra 2 nucleotides and the Kpn1 restriction enzyme site; Reverse primer contains the Not1 site. Restriction enzyme sites are highlighted and the additional two nucleotides are underlined.

Supplementary Table S3: Putative transcription factor binding motifs located in the 1000bp region upstream of the *SbGATA22* transcription start site (TSS).

Putative binding motif	Signal Pathway	Core Sequence	Position from TSS	Strand
Auxin Response Factor (ARF)	Auxin	GTCGG	-178<br>-319<br>-451	-
Dof	Light<br>Defense<br>C metabolism<br>Gibberellic acid<br>Auxin	AAAAG	-279<br>-863	+
AP2/Ethylene response factor				
GCC-Box	Ethylene	GCCGCC	-166<br>-175<br>-386	+
Drought response element (DRE)	Drought	CCGAC	-198<br>-450<br>-546	
MIKC-Type MADS-Box	Floral organ identity	CC(A/T)$_6$GG	-740	+

References

Chow, C.-N.; Zheng, H.-Q.; Wu, N.-Y.; Chien, C.-H.; Huang, H.-D.; Lee, T.-Y.; Chiang-Hsieh, Y.-F.; Hou, P.-F.; Yang, T.-Y.; Chang, W.-C. PlantPAN 2.0: an update of plant promoter analysis navigator for reconstructing transcriptional regulatory networks in plants. *Nucleic Acids Research* **2015**, *44*, D1154-D1160.
